# AGE AND GENDER DIFFERENCES IN ALCOHOL, TOBACCO, AND SUBSTANCE USE, REPRODUCTIVE HEALTH, AND AWARENESS OF SEXUALLY TRANSMITTED DISEASE PREVENTION AMONG SECONDARY SCHOOL STUDENTS IN BUKITTINGGI, INDONESIA

**DOI:** 10.21010/Ajidv19i2S.2

**Published:** 2025-10-17

**Authors:** NINDREA D. Ricvan, MING C. Long, HENDRIYANI Heni

**Affiliations:** 1 Department of Medicine, Faculty of Medicine, Universitas Negeri Padang, Bukittinggi, Indonesia, 26181; 2 Faculty of Medical and Life Sciences, Sunway University, Sunway City, Malaysia; 3 Department of Nutrition, Health Polytechnic of Semarang, Semarang, Indonesia, 50268

**Keywords:** Alcohol consumption, Cigarette smoking, Drug use, Adolescent reproductive health, STD awareness

## Abstract

**Background:**

The variations in behavioral health issues by sex and age among adolescents in Indonesia have not been comprehensively explored. This study aimed to describe age and gender based differences in alcohol consumption, tobacco and substance use, reproductive health knowledge, and awareness of sexually transmitted disease (STD) prevention among high school students in Bukittinggi, Indonesia.

**Materials and Methods:**

This study employed a school-based cross-sectional design conducted in Bukittinggi Municipality, West Sumatra Province, Indonesia, with a sample size of 254 secondary school students. We used multi-stage stratified clustered sampling technique to select the students, and asked the students to complete self-administered questionnaires during class on a regular school day. Data analysis was performed using descriptive statistics, and the Chi-square test.

**Results:**

The study revealed that while the majority had never used alcohol, tobacco, electronic cigarettes, and substances, there were still some former drinkers and smokers, as well as current smokers and electronic cigarette users, especially among males aged 14-15 years. A small percentage also reported using analgesics and energy drinks. The participants generally received education about AIDS or HIV at school, but there were some misconceptions and stigmatizing attitudes toward HIV-positive individuals. Health education regarding birth control was not universal. Awareness of safe sex or STD prevention methods varied by age and gender, with varying levels of knowledge about abstinence and condom use.

**Conclusion:**

We found slight variations among male and female students. The study findings suggested the need for sex-specific targeted interventions to improve behavioral health issues.

## Introduction

Smoking and substance use among adolescents are serious public health issues (Kugbey, 2023). In 2019, the Global Youth Tobacco Survey found that 19.2% of students aged 13-15 years smoked (WHO, 2021). In Indonesia, the prevalence of smoking among 10-18-year-olds has been increasing every year, from 7.2% in 2013 to 9.1% in 2018 (MoH, 2013; MoH, 2018). Similarly, the use of substances by youth, especially high school students, is rapidly increasing worldwide (Nawi *et al.*, 2021).

In Indonesia, a survey in 2021 found a lifetime substance use history prevalence of 1.93% (National Narcotics Agency, 2021). Furthermore, reproductive health and awareness of sexually transmitted diseases (STDs) prevention during adolescence influence sexual and reproductive behaviors and overall well-being during adulthood (Inthavong *et al.*, 2020). However, the World Health Organization (WHO) estimates that more than 1 million people are infected with sexually transmitted diseases daily, with half of them aged between 15 and 24 years (WHO, 2018). Despite the high prevalence, less than 15% of high school students in low and middle-income countries (LMICs) had comprehensive knowledge of STDs symptoms (Abdul *et al.*, 2018). In Indonesia, the 2018 Basic Health Survey found a low level of knowledge about STDs prevention, particularly prevention of HIV/AIDS, among a large proportion of high school students with only 28.6% having adequate knowledge (MoH, 2013).

Despite extensive global research on adolescent risk behaviors, there is limited data in the Indonesian context particularly concerning how these behaviors vary by age and sex within specific local settings. Most national surveys present aggregated data, overlooking regional and demographic nuances (Nindrea *et al.*, 2024a; Nindrea *et al.*, 2024b). Few studies in Indonesia have disaggregated adolescent risk behaviors across developmental stages or compared patterns between males and females. As a result, there is a lack of context-specific evidence to guide targeted health education, prevention programs, and policy development, especially in culturally distinct regions like West Sumatra (Nindrea *et al.*, 2024b; Nindrea *et al.*, 2024c). Addressing this gap is essential to designing effective, locally relevant interventions that align with adolescents’ specific needs and sociocultural environments.

Sex and age are known to influence both substance use and sexual behaviors. However, few studies have adequately described variations in risky behaviors by age and sex among adolescents in Indonesia. Bukittinggi, one of the municipalities in West Sumatra Province in Indonesia, is a diverse city with a growing population of adolescents. Empirical data regarding variations in risk behaviors by age and sex can provide insights for policy makers and provide baseline data for future programs and interventions. The objective of this study was to describe age and gender differences in alcohol use, tobacco use, substance use, reproductive health, and awareness of STDs prevention among secondary school students in Bukittinggi, Indonesia.

## Materials and Methods

### Study design and setting

A cross-sectional study was carried out within selected schools located in Bukittinggi Municipality, West Sumatra, Indonesia.

### Study participants and sample size calculation

The target population for this study consisted of secondary school students residing in Bukittinggi Municipality, West Sumatra, Indonesia. Eligibility criteria included students aged between 12 and 15 years who were literate in Bahasa Indonesia. Those who were absent during data collection or not currently enrolled in secondary school were excluded. The sample size was determined using a prevalence estimate of 21% for tobacco use (p = 0.21), with a 5% margin of error, resulting in a minimum required sample of 254 students (*Ahammed* et al., 2021).

### Sampling Techniques

This study employed a multistage stratified cluster sampling method. Initially, a comprehensive list of secondary schools was obtained from the Education Office of Bukittinggi Municipality, located in West Sumatra Province, Indonesia. From this list, two schools were randomly chosen. Within each selected school, classrooms were stratified by grade level, and two classrooms per grade were randomly selected based on the school-provided class list. All students within these chosen classrooms were invited to take part in the research.

### Operational definition

For age and sex distribution, participants were divided into four categories: (1) males aged 12–13 years, (2) females aged 12–13 years, (3) males aged 14–15 years, and (4) females aged 14–15 years. The age grouping was based on the typical age range of students at this educational level, which spans from 11 to 15 years and represents early adolescence (Nindrea *et al.*, 2024).

In terms of alcohol consumption, participants were classified into three groups: (1) those who had never consumed alcohol (lifetime abstainers), (2) those who had consumed alcohol at some point in their lives but not within the last 12 months (former drinkers), and (3) those who had consumed alcohol within the past 12 months (current drinkers) (Robert Lourdes *et al.*, 2022). Similarly, tobacco use was categorized as follows: (1) individuals who had never smoked or had smoked fewer than 100 cigarettes in their lifetime (never smokers), (2) individuals who had smoked 100 or more cigarettes in their lifetime but had not smoked in the past 30 days (former smokers), and (3) individuals who had smoked within the past 30 days (current smokers) (Dube *et al.*, 2019). For electronic cigarette use, we decided to categorize the respondents as: 1) never users (never used electronic cigarette in lifetime); 2) former users (used electronic cigarette in lifetime, but not in past 30 days); 3) current users (used electronic cigarette in past 30 days) (Kim *et al.*, 2020).

For substance use, we categorized the respondents based on their lifetime history of substance use (Wahab *et al.*, 2021). In assessing awareness regarding prevention of sexually transmitted diseases (STDs), we evaluated knowledge about reproductive health and STDs prevention, as well as their attitudes towards and discrimination against HIV-positive individuals using a Likert scale (Folasayo *et al.*, 2017). The questions used to measure prevention of STDs awareness underwent validity and reliability testing, with a Cronbach’s alpha value >0.7.

### Data collection technique

Trained data collectors visited the selected schools and presented an official letter outlining the study’s objectives and procedures to school authorities. They then scheduled a suitable time for data collection and briefed students on the study’s purpose, their right to decline participation, the voluntary nature of involvement, and the measures taken to protect their privacy and confidentiality. Verbal consent was obtained from each student prior to participation.

Participants completed a self-administered questionnaire, sealed it in an individual envelope, and submitted it to a designated collection area within the classroom. The study coordinators later gathered all envelopes from each class and compiled them accordingly. Research personnel then retrieved the responses, reviewed them for completeness and consistency, and entered the verified data into a secure electronic database.

The institutional review board (IRB) granted a waiver of written parental consent to safeguard the students’ anonymity and uphold their confidentiality. Instead, students received verbal explanations of the study and were asked to provide informed verbal consent prior to participation.

### Data management

Completed paper questionnaires were digitized using the KoboToolbox platform by a team of trained personnel. For quality assurance, the research team cross-checked each participant’s unique identification number and survey completion timestamp from the metadata against the corresponding ID on the informed consent form. Any inconsistencies identified were addressed daily to ensure the data set was complete and accurately reflected participant information.

### Ethical considerations

Ethical clearance for this study was granted by the Ethics Committee of Dr. M. Djamil Central General Hospital in Padang, under reference number DP.04.03/D.XVI.X/537/2023.

### Data analysis

Descriptive statistics were utilized to summarize the demographic and behavioral profiles of the participants. The students were categorized into four distinct groups based on age and gender: males aged 12–13, females aged 12–13, males aged 14–15, and females aged 14–15. For the bivariate analysis, the Chi-square test was employed to examine associations between categorical variables. Statistical significance was determined at a threshold of p < 0.05. All data analyses were performed using R version 4.3.0.

## Results

Characteristics of the study participants are as shown in Table 1.

**Table 1:** Characteristics of respondents

Characteristic		Frequency (%)
**Ethnicity**		
	Minangnese	145 (57.1)
	Javanese	37 (14.6)
	Bataknese	9 (3.5)
	Sundanese	4 (1.6)
	Others	59 (23.2)
**Father’s occupation**		
	Civil servant	53 (20.9)
	Private sector employee	94 (37.0)
	Small-scale vendors	41 (16.1)
	Entrepreneur	27 (10.6)
	Laborer	19 (7.5)
	Agriculture/ fishery	17 (6.7)
	Independent professions (e.g., lawyers, architects)	3 (1.2)
	Father’s education	
	Junior high school	37 (14.6)
	Senior high school	88 (34.6)
	Vocational certificate	2 (0.8)
	Associate’s degree	70 (27.6)
	Bachelor’s degree	52 (20.5)
	Higher than bachelor’s degree	5 (1.9)
**Mother’s occupation**		
	Housewife	173 (68.1)
	Civil servant	16 (6.3)
	Private sector employee	21 (8.3)
	Small-scale vendors	40 (15.7)
	Entrepreneur	4 (1.6)
**Mother’s education**		
	Primary school	2 (0.8)
	Junior high school	45 (17.7)
	Senior high school	102 (40.2)
	Vocational certificate	4 (1.6)
	Associate’s degree	80 (31.5)
	Bachelor’s degree	21 (8.3)
**Household monthly income (IDR)**		
	< 1,000,000	0
	1,000,000 to 2,000,000	31 (12.2)
	2,000,001 to 3,000,000	61 (24.0)
	3,000,001 to 4,000,000	96 (37.8)
	4,000,001 to 5,000,000	47 (18.5)
	5,000,001 to 6,000,000	19 (7.5)
	Religion	
	Islam	241 (94.9)
	Christianity	13 (5.1)

Table 1 presents the demographic characteristics of the participants, revealing that the majority were of Minangnese ethnic background and identified as Muslim. A large proportion of students reported that their fathers worked in the private sector and had completed senior high school, while their mothers were primarily homemakers with a similar level of education. The most frequently reported household monthly income ranged from 3,000,001 to 4,000,000 IDR. Comparison of alcohol use, tobacco use, substance use, reproductive health and STDs prevention awareness by sex and age among secondary school students in Bukittinggi, Indonesia (Table 2).

Table 1 presents the demographic characteristics of the participants, revealing that the majority were of Minangnese ethnic background and identified as Muslim. A large proportion of students reported that their fathers worked in the private sector and had completed senior high school, while their mothers were primarily homemakers with a similar level of education. The most frequently reported household monthly income ranged from 3,000,001 to 4,000,000 IDR.

Comparison of alcohol use, tobacco use, substance use, reproductive health and STDs prevention awareness by sex and age among secondary school students in Bukittinggi, Indonesia (Table 2).

**Table 2:** Comparison of alcohol use, tobacco use, substance use, reproductive health and STDs prevention awareness by sex and age among secondary school students in Bukittinggi, Indonesia

Behavior	Male, 12-13 years (n=33)	Female, 12-13 years (n=51)	Male, 14-15 years (n=73)	Female, 14-15 years (n=97)	p-value
**Alcohol use**					
Lifetime abstainers (never drank alcohol)	33 (100.0)	51 (100.0)	70 (95.9)	97 (100.0)	0.057
Former drinkers (drank alcohol in lifetime, but not in past 12 months)	0	0	3 (4.1)	0	
Current drinkers (drank alcohol in past 12 months)	0	0	0	0	
**Tobacco use**					
Never smokers (never smoked or smoked less than 100 cigarettes)	32 (97.0)	50 (98.0)	62 (84.9)	95 (97.9)	0.001*
Former smokers (smoked 100 cigarettes or more in lifetime, but not in past 30 days)	0	1 (2.0)	0	1 (1.0)	
Current smokers (smoked in past 30 days)	1 (3.0)	0	11 (15.1)	1 (1.0)	
**Electronic cigarette use**					
Never users (never used electronic cigarette in lifetime)	32 (97.0)	51 (100.0)	65 (89.0)	95 (97.9)	0.054
Former users (used electronic cigarette in lifetime, but not in past 30 days)	0	0	2 (2.7)	0	
Current users (used electronic cigarette in past 30 days)	1 (3.0)	0	6 (8.2)	2 (2.1)	
**Lifetime history of substance use (yes)**					
Analgesic (not as medication) (parasetamol)	1 (3.0)	0	2 (2.7)	0	0.235
Energy drinks (extra joss, M-150, kratingdaeng, hemaviton, etc)	4 (12.1)	2 (3.9)	7 (9.6)	2 (2.1)	0.071
All other substances**	0	0	0	0	n/a
**Prevention of Sexually Transmitted Diseases (STDs)**					
**Taught about AIDS or HIV at school (yes)**					n/a
Never	0	0	0	0	
Yes	33 (100.0)	51 (100.0)	73 (100.0)	97 (100.0)	
Don’t know	0	0	0	0	
Refuse to answer	0	0	0	0	
**Agree or strongly agree:** I do not want to give salaam when greeting someone who is HIV/AIDS positive	2 (6.1)	1 (2.0)	0	5 (5.2)	0.184
**Agree or strongly agree:** Adolescents who are HIV-positive should be banned from attending schools with HIV-negative students	2 (6.1)	1 (2.0)	0	5 (5.2)	0.187
**Agree or strongly agree:** Adolescents who are HIV-positive should not swim in a pool with HIV-negative students	2 (6.1)	3 (5.9)	0	5 (5.2)	0.233
**Agree or strongly agree:** Adolescents who are HIV-positive should not take part in sports competitions with HIV-negative students	2 (6.3)	1 (2.0)	0	5 (5.2)	0.181
**Reproductive Health**					
**Taught about birth control at school**					0.182
Never	3 (9.1)	1 (2.0)	1 (1.4)	3 (3.1)	
Yes	30 (90.9)	50 (98.0)	72 (98.6)	94 (96.9)	
Don’t know	0	0	0	0	
Refuse to answer	0	0	0	0	
**Ever had a romantic partner (yes)**	8 (24.2)	5 (9.8)	13 (17.8)	13 (13.4)	0.280
**Method of safe sex or STD prevention known to participant (multiple answers allowed)**					
None	0	0	0	0	n/a
Abstinence	9 (27.3)	11 (21.6)	17 (23.3)	9 (9.3)	0.034*
Condom	33 (100.0)	51 (100.0)	72 (98.6)	93 (95.9)	0.245
All other methods***	0	0	0	0	n/a

a For Chi-square test;

* bold number with asterisk denotes statistical significance at 95% level of confidence; n/a, not applicable

** Other substances included: 1) Antihistamine (not as medication); 2) Cough syrup (not as medication); 3) Anxiolytics; 4) Sedatives; 5) PRO [procodyl, promethazine]; 6) LEAN [purple drank]; 7) Poppers; 8) Kratom leaf; 9) Kratom leaf tea mixed with other substance; 10) Cannabis; 11) Opium; 12) Ecstasy / Love Drug (Inex); 13) Ketamine; 14) Heroin; 15) Inhalants (paint thinner, glue, benzene); 16) Methamphetamine; 17) Crystal methamphetamine

*** All other methods included: 1) Avoiding activities high risk with mucosal tear; 2) Suppository; 3) Taking Pre-exposure prophylaxis (PreP); 4) Taking Post-exposure prophylaxis (PEP); 5) Other methods

Comparison of alcohol use, tobacco use, substance use, and awareness of reproductive health and prevention of sexually transmitted diseases by sex and age (Table 2) showed that almost none of the participants had used alcohol or drugs in their lifetime, except for very few 14-15 years old boys. However, use of smoking tobacco and electronic cigarettes was higher among boys than girls, with boys age 14-15 years having particularly high prevalence. All participants were taught about HIV/AIDS in the school. In all age groups, participants reported a tolerant and accepting attitude towards peers infected with HIV. Over 90 percent of the participants in all groups were taught about birth control. However, boys were significantly more likely to report awareness of abstinence as a method for safe sex. Nearly all participants reported awareness of condom for safe sex. However, no one reported awareness regarding PrEP and PEP.

## Discussion

In this school-based cross-sectional study, we described variations by age and sex in substance use, HIV-related attitude, and STD prevention awareness among secondary school students in Bukittinggi, Indonesia. We found that boys were significantly more likely to report smoking tobacco and using electronic cigarettes than girls, particularly those aged 14-15 years. However, almost no one reported using alcohol or illicit drugs. Boys were more likely to report abstinence and condom as a method for STD prevention than girls. However, no one was aware of pre-exposure and post-exposure prophylaxes. The findings of this study have implications for relevant stakeholders as basic information to inform resource allocation and as baseline information for future programs.

This gender difference in tobacco and substance use behaviors highlights the need for gender-specific interventions to address these issues. These findings are consistent with a prior study conducted in Iran, which documented an overall smoking prevalence of 9.8%. The study also identified a significant gender gap, with smoking rates of 17.6% in males and 4.2% in females (Taheri *et al.*, 2014). Another study among junior high school students found that 15.2% of males and 3.5% of females had smoked in the past 12 months (Nindrea *et al.*, 2024). A study conducted in Japan on secondary school students indicated that substance use was more prevalent among males than females due to the influence of social circles (Miyoshi *et al.*, 2012).

This finding has implications for the timing and targeting of interventions aimed at promoting healthy behaviors and increasing awareness of reproductive health and STD prevention among adolescents. Male students exhibited better awareness of STD prevention compared to their female counterparts. The findings of our study were similar to those from a study in Cape Town, South Africa, which found that male students’ perceptions of STDs were higher at 54.6% compared to females at 45.4% in grades 6-12 (Nyasulu *et al.*, 2018; Hendriyani *et al.*, 2020). This could happen due to differences in socialization patterns, or even variations in the educational curriculum for each grade regarding STDs. Additionally, societal attitudes towards gender roles and sexuality could also have influenced the formation of these perceptions differently for male and female students (Nindrea *et al.*, 2020). This finding underscores the importance of understanding gender-specific attitudes and knowledge regarding reproductive health and STD prevention (Nindrea *et al.*, 2019).

In this study, it was found that students had tolerance towards attitudes and discrimination against HIV-positive individuals. This was attributed to all students having received education about AIDS or HIV at school. Additionally, the findings could indeed have been subject to social desirability bias, where participants may have responded in a way that they believed was socially acceptable rather than expressing their true attitudes or behaviors. Furthermore, the research found a lack of awareness regarding PrEP and PEP. Therefore, there is a need for the promotion of PrEP and PEP awareness (or otherwise) among adolescents.

The practical implications of these findings are multifaceted. First, health education curricula in secondary schools should be revised to ensure they address not only general sexual health but also specific, evidence-based information on HIV prevention strategies such as PrEP and PEP. Second, gender-sensitive health promotion campaigns are needed to tailor messages and interventions to both male and female students, recognizing the distinct risk profiles and information gaps between them. Third, integrating family and community-based education may be effective in reinforcing school-based efforts, particularly in culturally conservative areas. Lastly, policymakers and health authorities could use these findings to prioritize early intervention and targeted programs in urban centers like Bukittinggi City, thereby optimizing resource allocation for adolescent health promotion. These actions could contribute to reducing risk behaviors and improving long-term health outcomes for Indonesian youth.

The strength of this study lies in the self-administration of the study questionnaire, which helps to reduce social desirability bias, to an extent. Nonetheless, several limitations must be taken into account when interpreting the results of this study. Firstly, the cross-sectional design only allows for the description of variations in risky health behaviors at a single point in time. Our participants may engage in more or less risk behaviors during their life course. We also asked about lifetime history of behaviors, which may be subjected to recall errors. Lastly, questions regarding attitude towards HIV infected peers may be subject to response acquiescence or self-serving biases and may not reflect real-world processes.

## Conclusion

In conclusion, this study reveals notable differences between male and female secondary school students in Bukittinggi, Indonesia, particularly in tobacco smoking, e-cigarette use, and awareness of STDs prevention. These findings offer valuable baseline data that can inform policymakers, educators, and public health stakeholders in developing targeted interventions to address adolescent health risks.

### Conflict of Interest

The authors declare that there is no conflict of interest associated with this study.

**List of Abbreviations:**
AIDS: Acquired immune deficiency syndrome;
HIV: Human immunodeficiency virus;
PEP: Post-exposure prophylaxis;
PreP: Pre-exposure prophylaxis;
STDs: Sexually transmitted diseases
